# Factors associated with diagnostic delay of pulmonary tuberculosis among children and adolescents in Quzhou, China: results from the surveillance data 2011–2021

**DOI:** 10.1186/s12879-023-08516-1

**Published:** 2023-08-18

**Authors:** Yating Zhang, Bingdong Zhan, Xiaogang Hao, Wei Wang, Xing Zhang, Chunfu Fang, Min Wang

**Affiliations:** 1 School of Public Health, Zhejiang University of Traditional Chinese Medicine, Hangzhou, Zhejiang China; 2 Department of Tuberculosis Control and Prevention, Quzhou Center for Disease Control and Prevention, No.154, Xi‘an Road, Ke Cheng District, Quzhou, Zhejiang 324000 China

**Keywords:** Pulmonary tuberculosis, Delay, Children, Patient delay, Health system delay

## Abstract

**Purpose:**

Tuberculosis is a high-burden disease and a major health concern in China, especially among children and adolescents. The purpose of this study was to assess risk factors for diagnostic delay in students with pulmonary tuberculosis in Quzhou City in eastern China.

**Patients and methods:**

Cases of PTB in students and relevant information in Quzhou from 2011 to 2021 were collected using the TB Management Information System. The outcome of interest was diagnostic delay (i.e. ≥ 28 days between symptom onset and treatment initiation). Risk factors for diagnostic delay were identified using multivariable logistic regression.

**Results:**

A total of 629 students in Quzhou were diagnosed with PTB during the study period, of whom 55.5% were male. The median diagnostic delay was 18 days (Inter Quartile Range, [IQR]: 8–38) and 38.0% of the students had a diagnostic delay. Living in a rural area (adjusted odds ratio, [AOR]: 1.56, 95% confidence interval [CI:] 1.11–2.19), developing PTB symptoms in the first quarter of the year (AOR: 2.18, 95% CI: 1.40–3.40), and no sputum smear result (AOR: 8.73, 95% CI: 1.68–45.30) were significantly associated with a diagnostic delay. Discovery through health examinations (AOR: 0.33, 95% CI: 0.17–0.63) was associated with reduced risk of diagnostic delay.

**Conclusion:**

Schools in rural areas should pay special attention to increasing student awareness of the symptoms of tuberculosis and provide health education on tuberculosis prevention and control to students and staff.

## Introduction

Tuberculosis is a chronic infectious disease caused by *Mycobacterium tuberculosis*, which often invades pulmonary. Until the Corona Virus Disease 2019 (COVID-19) pandemic, tuberculosis was the top cause of death from a single infectious agent. According to World Health Organization Global Tuberculosis Report 2022 [1], an estimated 10.6 million people became ill with tuberculosis and 1.6 million people died in 2021, with an incidence of 134 cases per 100,000 population and a mortality of 17 cases per 100,000 population globally. Globally, China ranks second among the 30 countries with a high PTB burden in 2021. The national PTB notification rate (new and relapsed cases) in 2021 is 55 per 100,000 population/year [1].

PTB is a major health problem that is characterized by high risk, insidious transmission and difficult to manage. If not untreated, PTB can cause serious health risks such as systemic symptoms of poisoning, spread and infection of tuberculosis, proliferation of tubercle bacilli, permanent lung damage and other adverse effects. Limited knowledge about PTB signs and symptoms, poor health-seeking behavior, and poor diagnosis and disease management in health facilities have resulted in delays in PTB diagnosis and treatment. Delays in diagnosis and detection have been identified as one of the key challenges to eliminate tuberculosis [2]. Most transmissions occur between the appearance of cough and initiation of treatment. Madebo et al [3] found that patients become more contagious as the delay progresses. The contagion parameter suggests that where tuberculosis is endemic, each infectious case will result in between 20 and 28 secondary infections [4]. Delays in PTB diagnosis and treatment in turn may worsen the disease, result in more complications, lead to a higher mortality rate and increase the development of multidrug-resistant tuberculosis. Once suffering from multidrug-resistant tuberculosis, the proportion of patients receiving standardized treatment will decline. The cost of treatment has been a major barrier as it is 100-times more expensive to treat multidrug-resistant tuberculosis as opposed to drug-susceptible tuberculosis due to the number of medications and clinical management of its prolonged and potentially toxic treatment course [5, 6]. Therefore, early diagnosis and immediate treatment are one of the effective ways to prevent the community transmission of tuberculosis.

In China, PTB case-finding is passive, and depends on patients presenting to health facilities with tuberculosis symptoms. Although the incidence rate of tuberculosis among students is lower (13.64 cases per 100,000/year in 2021 [7]) than the general population(55 cases per 100,000/year in 2021 [1]), students live in dense settings that are prone to tuberculosis outbreaks and public health emergencies. In addition, if students with PTB are not promptly diagnosed and treated, the widespread transmission of *M. tuberculosis* can occur because schools provide several avenues for tuberculosis transmission [8]. In 2020, the Chinese government issued *guidelines for the prevention and control of tuberculosis in schools in China* [9], which requires that a network of school tuberculosis prevention and control work, consisting of administrative departments such as health and education works together to do a good job of preventing and controlling tuberculosis in schools. In the same year, Quzhou began to implement a zero-burden treatment cost pilot program for ordinary tuberculosis and drug-resistant tuberculosis, and was the first prefecture-level city in the country to introduce a policy of fully subsidizing the costs of diagnosis and treatment of rifampicin-resistant tuberculosis and ordinary tuberculosis. But the incidence of tuberculosis among students in Quzhou was 24.07 cases per 100,000 person in 2012 [10], higher than the national average incidence rate of 15.02 cases per 100,000/year among students in the same year. There may be some undetected factors influencing the incidence of tuberculosis among students. Thus, in this study, we aimed to assess the risk factors for diagnostic delay of PTB among students in order to prevent outbreaks and provide information to promote PTB control strategies in students.

## Methods

### Study area

**Fig. 1:**
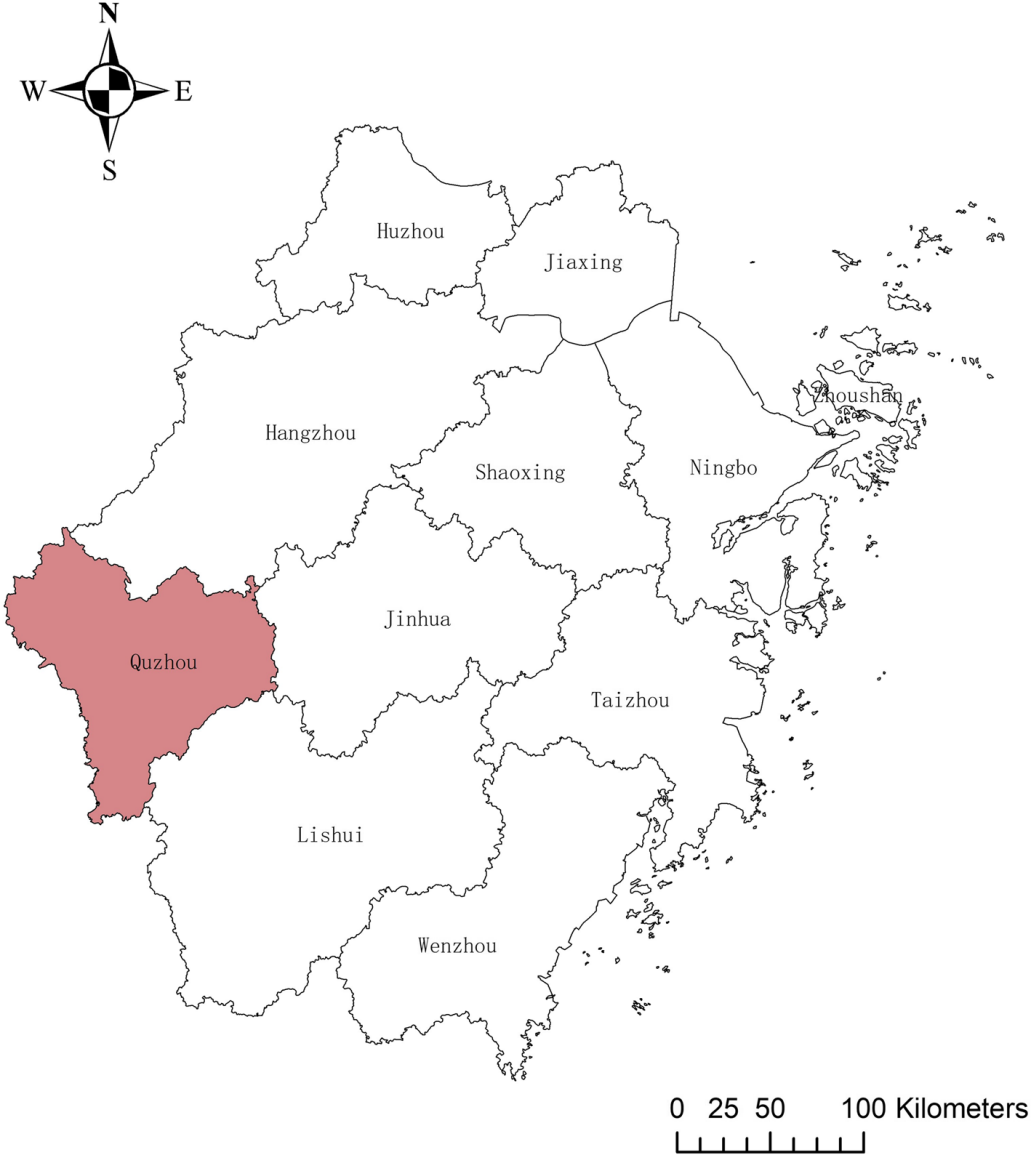
Location of Quzhou City in Zhejiang

Quzhou City is located in the western region of the Zhejiang Province in China and has a land area of 8844 km^2^ and a gross domestic product of 1876 trillion RMB in 2021. The city consists of six counties (Kecheng, Qujiang, Jiangshan, Longyou, Changshan, and Kaihua. Quzhou), and has a subtropical monsoonal climate and four distinct seasons, with sufficient light and heat, abundant rainfall, and moderate temperatures. The rivers in Quzhou belong to the Qiantang River System. The watershed covers 8332.9 km^2^, accounting for 94.2% of the total land area. The location of Quzhou City is shown in Fig. 1.

### Data source

Data on all PTB cases among students in Quzhou City were collected from the Tuberculosis Information Management System (TBIMS) between January 2011 and December 2021. We excluded cases of only extrapulmonary tuberculosis and nontuberculous mycobacterial infection. The study included kindergarten, primary school, high school, and university students. The details of each PTB case included basic demographic information, clinical diagnostic information, laboratory test results, and treatment classification. The annual student population data were obtained from the Quzhou Statistical Yearbook.

### Definitions

Children aged 3–6, 7–12, 13–15, and 16–18 years were defined as kindergarten, primary school, junior high school, and senior high school students, respectively. Adults aged 19–25 years were classified as college students or above. This study identified PTB cases included laboratory-confirmed and clinically diagnosed cases. Clinically diagnosed PTB cases were defined as those with PTB-specialized chest imaging, clinical manifestations (coughing, expectoration for ≥ 2 weeks, or hemoptysis), and no response to diagnostic anti-inflammatory therapy with negative laboratory test results or missing results (anti-TB drug was excluded) [11], especially cases in children under five years of age should be accompanied by imaging abnormalities and clinical manifestations as well as a positive tuberculin skin test or a positive INF-γ interferon release test [12]. Laboratory confirmation of PTB was based on sputum smear, culture, and GeneXpert results indicating infection with M. tuberculosis. Smear-positive PTB were defined the patients with two or more sputum smears for acid fast bacilli (AFB) or one sputum positive for AFB and radiological abnormalities consistent with active PTB. Smear-negative PTB were defined the patients with three negative sputum smears for AFB and radiological abnormality consistent with active PTB or failure to respond to antibiotics trials [13]. Patient delay was defined as the time interval between the date of PTB symptom(i.e. persistent cough, fever, weakness, weight loss or chest pain) onset to the first visit to a professional healthcare provider. Health system delay referred to the time between the date of the first presentation of the patient to a professional healthcare provider to the initiation of treatment. The total diagnostic delay (hereafter referred to as diagnostic delay) was defined as the sum of the patient and health system delays [14]. Based on previous studies, we used 28 days as the cutpoint for analyzing diagnostic delay and 14 days as the cutpoint for analyzing patient and health system delays [15, 16].Resident population was defined as living at the research site for at least 6 months. Migrant population refers to have left their place of domicile to live in Quzhou for various reasons [17]. Migrant population generally do not have local medical security, and cannot be reimbursed after seeking medical treatment. They can only enjoy the free anti-tuberculosis compound medication provided by the country. Urban residents are based on the place of residence, those who live in towns and cities above the county level, while the rest are categorized as rural residents. The first quarter of the year is from March through May.The second quarter is from June through July. The third quarter is from September through November. The forth quarter is from December through February. According to Technical Guidelines for Tuberculosis Control in China [18], passive finding refers to the patient's initiative to go to tuberculosis prevention and control institutions for treatment due to symptoms. Referral refers to non designated hospitals and non tuberculosis out-patient clinics of designated hospitals, where patients with suspicious symptoms are checked, and tuberculosis or suspected tuberculosis patients are transferred to tuberculosis out-patient clinics of tuberculosis designated medical institutions in a timely manner. Tracking refers to the CDC's tracking of suspected patients who are not referred in a timely manner. Symptoms referral means that community and village doctors organize or refer people with suspected symptoms of tuberculosis to the tuberculosis control facility for examination. Active screening refers to screening for tuberculosis in high-risk groups such as close contacts of pathologically positive tuberculosis patients, HIV-infected patients and AIDS patients. Health examination means that patients with tuberculosis and suspected tuberculosis detected by means of health check-ups conducted by medical institutions.

### General characteristics of PTB in students

Data were collected on sex, age group, current residence, registered residence, treatment classification, date of onset of TB-related symptoms, TB discovery method, and centre of first contact.

#### Statistical analysis

We used descriptive statistics to describe the participants’ characteristic and Pearson’s χ^2^ test or Fisher’s exact test to determine differences in the diagnostic delay proportions and evaluate the annual percentage changes. Variables that were significantly associated with a diagnostic delay in the univariable analyses and of epidemiological interest were included in the multivariable logistic regression model, with the estimation of their adjusted odds ratios (AORs) and 95% confidence intervals (CIs). We set the criteria for entering and exiting the model to P < 0.05 and P > 0.10, respectively. Two-sided P values of < 0.05 were considered statistically significant. Descriptive analyses were conducted using SPSS for Windows (version 25.0; IBM Corp, Armonk, NY, USA) and Microsoft Excel (Microsoft Corporation, Redmond, WA, USA). The significance of changes in the annual diagnostic delay rates was assessed using the chi-squared test for trend. Related figure were plotted using the ArcGIS software (version 10.5; ESRI Inc., Redlands, CA, USA).

## Results

### General characteristics of PTB in students

**Fig. 2:**
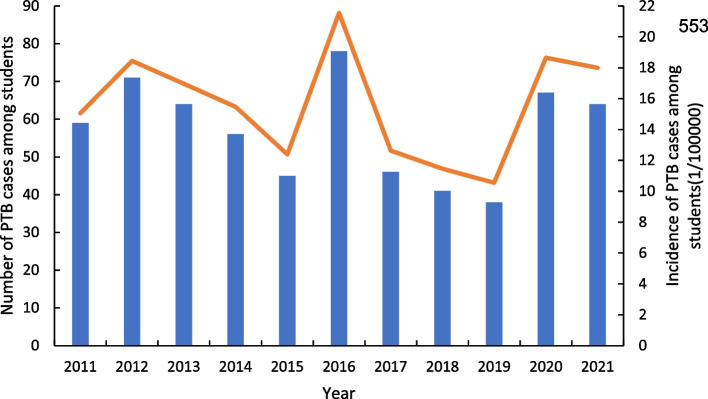
Trend of PTB incidence and number of PTB cases by year. Abbreviation: PTB, pulmonary tuberculosis

A total of 629 cases of PTB in student were reported in Quzhou City between 2011 and 2021, accounting for 0.2% of all (*n* = 363,736) students. The incidence of PTB among students ranged from 10.55 to 21.55 cases per 100,000/year, with an average annual incidence rate of 18.60 cases per 100,000/year (Fig. 2). Of the students diagnosed with TB, 55.5% (*n* = 349) were male. The age distribution was as follows: 11.9% (*n* = 75) were 13–15 years, 52.8% (*n* = 332) were 16–18 years, and 31.3% (*n* = 197) were 19–25 years. Regarding registered residences, 98.3% (*n* = 618) were residents. Concerning current residence, 51.5% (*n* = 324) were from rural areas.. Regarding the appearance of TB-related symptoms, 32.3% (*n* = 203) became symptomatic in the first quarter of the year. In terms of diagnostic results, 16.2% (*n* = 102) were positive sputum-smear, 8.7%(*n* = 55) were positive sputum-culture, 7.6%(*n* = 48) were positive molecular biology. 99.5%(*n* = 626) presented as an imaging abnormality.; 99.2% of the PTB cases were initially treated, and 40.4% were identified through referral and tracing. County-level designated hospitals (65.5%) were the type of healthcare facility sought after the onset of symptoms (Table 1).

**Table 1:** Evaluation of factors associated with diagnostic delay in students in Quzhou with pulmonary tuberculosis using univariable analysis

Variable	Total PTB (*N* = 629)	Diagnostic delay (*N* = 239)	No delay (*N* = 390)	*P* value
**Sex, n (%)**				0.21
Male	349 (55.5)	125 (52.3)	224 (57.4)	
Female	280 (44.5)	114 (47.7)	166 (42.6)	
Grade (age in years), n (%)*				0.084
Kindergarten (3–6)	3 (0.5)	2 (0.8)	1 (0.3)	
Primary school (7–12)	22 (3.5)	10 (4.2)	12 (3.1)	
Junior high school (13–15)	75 (11.9)	36 (15.1)	39 (10.0)	
Senior high school (16–18)	332 (52.8)	112 (46.9)	220 (56.4)	
College and above (19–25)	197 (31.3)	79 (33.0)	118 (30.3)	
**Registered residence, n (%)**				0.41
Resident population	618 (98.3)	233 (97.5)	385 (98.7)	
Migrant population	11 (1.7)	6 (2.5)	5 (1.3)	
**Current residence, n (%)**				0.01
Rural	324 (51.5)	138 (57.7)	186 (47.7)	
Urban	305 (48.5)	101 (42.3)	204 (52.3)	
**TB-related symptoms appearance time, n (%)**				< 0.001
First quarter of the year	203 (32.3)	99 (41.4)	104 (26.7)	
Second quarter of the year	185 (29.4)	55 (23.0)	130 (33.3)	
Third quarter of the year	123 (19.6)	45 (18.8)	78 (20.0)	
Fourth quarter of the year	118 (18.7)	40 (16.8)	78 (20.0)	
**Sputum smear, n(%)**				0.013
Positive	102 (16.2)	45 (18.8)	57 (14.6)	
Negative	518 (82.4)	187 (78.3)	331(84.9)	
Not tested/not available	9 (1.4)	7 (2.9)	2 (0.5)	
**Treatment classification, n (%)**				> 0.99
First treatment	624 (99.2)	237 (99.2)	387 (99.2)	
Retreatment	5 (0.8)	2 (0.8)	3 (0.8)	
**Patient discovery method, n (%)**				< 0.001
Referral and tracing	254 (40.4)	94 (39.3)	160 (41.0)	
Passive finding	233 (37.1)	97 (40.6)	136 (34.9)	
Symptomatic referral	33 (5.3)	13 (5.4)	20 (5.1)	
Health examination	78 (12.4)	15 (6.3)	63 (16.2)	
Active screening	31 (4.9)	20 (8.4)	11 (2.8)	
**Centre of first contact, n(%)**				0.438
County medical institutions	412 (65.5)	152 (63.6)	260 (66.7)	
Municipal medical institutions	217 (34.5)	87 (36.4)	130 (33.3)	

### *Diagnostic delay of pulmonary tuberculosis patients before and after the COVID-19 epidemic*

A total of 279 students (44.4%) experienced patient delay, whereas there was a low incidence of health system delay (*n* = 85, 13.5%). The median diagnostic delay was 18 days (IQR: 8–38), and the incidence of diagnostic delay ranged from 29.7% to 59.0%, with an annual average of 38.0% (Table 2). There was no statistically significant difference in the diagnostic delay before and after the COVID-19 epidemic (*P* = 0.17).

**Table 2:** Delayed diagnosis of pulmonary tuberculosis patients in different years

Year	**Total PTB**(*N* = 629)	Diagnostic delay(*N* = 239)
Before the COVID-19 epidemic(%)		
**2011**	**59**	**25(42.4)**
**2012**	**71**	**23(32.4)**
**2013**	**64**	**20(31.3)**
**2014**	**56**	**20(35.7)**
**2015**	**45**	**18(40.0)**
**2016**	**78**	**46(59.0)**
**2017**	**46**	**17(37.0)**
**2018**	**41**	**14(34.1)**
**2019**	**38**	**13(34.2)**
Subtotal	**498**	**196(39.4)**
After the COVID-19 epidemic(%)		
**2020**	**67**	**24(35.8)**
**2021**	**64**	**19(29.7)**
**Subtotal**	**131**	**43(32.8)**
**Total**	**629**	**239(38.0)**

### Factors associated with a diagnostic delay in the* univariable analysis*

Univariable analysis revealed differences in current residence, TB-related symptom appearance time, diagnostic test results, and patient discovery method between patients who experienced diagnostic delay and other patients. The incidence of diagnostic delay in students living in rural areas (57.7%) was significantly higher than that in students living in urban areas (42.3%) (*P* = 0.01). The incidence of diagnostic delay in students who developed PTB symptoms in the first quarter of the year(41.4%) was higher than that in the other quarters (*P* < 0.001). The incidence of diagnostic delay was higher among students with negative sputum-smear (78.3%) than among students with positive sputum-smear disease (18.8%) or not (2.9%) tested (*P* = 0.013). The incidence of diagnostic delay in students found through passive discovery (40.6%) was significantly higher than that through other discovery methods (*P* < 0.001) (Table 1).

### Factors associated with a diagnostic delay in the multivariable logistic regression analysis

Multivariable logistic regression analysis showed that living in a rural area ( AOR: 1.56, 95% CI: 1.11–2.19), presenting with PTB symptoms in the first quarter of the year (AOR: 2.18, 95% CI: 1.40–3.40), and diagnosing as not available sputum-smear (AOR: 8.73, 95% CI: 1.68–45.30) were significantly associated with a diagnostic delay of ≥ 28 days. In contrast, discovery through health examinations (AOR: 0.33, 95% CI: 0.17–0.63) was associated with reduced risk of diagnostic delay (Table 3).

**Table 3:** Evaluation of factors associated with diagnostic delay occurrence in PTB cases among students in Quzhou using multivariate logistic regression

Variable	AOR (95% CI)	*P* value
**Current residence**		
Urban	1.00 (reference)	
Rural	1.56 (1.11–2.19)	0.011
**TB-related symptoms appearance time**		
Second quarter of the year	1.00 (reference)	
First quarter of the year	2.18 (1.40–3.40)	0.001
Third quarter of the year	1.28 (0.77–2.11)	0.338
Fourth quarter of the year	1.04 (0.62–1.74)	0.885
**Sputum smear**		
Negative	1.00 (reference)	
Positive	1.39 (0.89–2.18)	0.145
Not tested/not available	8.73 (1.68–45.30)	0.010
**Patient discovery method**		
Passive case-finding	1.00 (reference)	
Health examination	0.33 (0.17–0.63)	0.001
Referral and tracing	0.85 (0.58–1.24)	0.398
Symptomatic referral	0.63 (0.28–1.39)	0.250
Active screening	2.02 (0.90–4.55)	0.089

*Abbreviations: AOR* Adjusted odds ratio, *CI* Confidence interval, *TB* Tuberculosis

## Discussion

Although a preventable and treatable disease, the TB disease burden in Quzhou CIty is high, with a high annual incidence rate [9]. Our study used conventional epidemiological methods to determine the incidence and length of diagnostic delay among students diagnosed with PTB in Quzhou, Zhejiang Province. Understanding the risk of diagnostic delay among this high-risk group could provide a theoretical basis for optimizing school TB prevention and control measures.

In our study, the median diagnostic delay among students with PTB (18 days), was lower than that reported for all PTB patients in Argentina (58 days) [19], Ethiopia (70.5 days) [14], New York (57 days) [20], France (68 days) [21], Norway (63 days) [22], and Türkiye (49 days) [23]. In addition, the mean annual incidence of diagnostic delay was 38.0%, which was moderate compared with the results of other studies conducted in China [24–26]. Differences in the diagnostic delay period between these studies are probably due to the heterogeneity in the study design and sample size; PTB incidence rate; sociodemographic, cultural, and economic status of the study populations; and the period in which the studies were conducted [27]. Moreover, mass admission screening for PTB among students may have effectively reduced the number of students with PTB entering school and the interval between symptom onset and treatment initiation, potentially playing a vital role in the early identification of active PTB cases [28].

In China, about 80% of TB patients live in rural areas [29]. In China’s 2010 National TB Prevalence Survey, the prevalence of active PTB in rural areas was about 1.8 times that of urban areas; that of sputum smear-positive TB was about 1.6 times that of urban areas. This large population of TB patients has a high prevalence of anti-TB drug resistance, which increases the rate of treatment failure and costs of control, and is a major challenge to public health for China [30]. Our results showed that students living in rural areas were more likely to experience a diagnostic delay in PTB, consistent with previous studies conducted elsewhere [31–34]. This implies that, in rural areas, long delays for rural residents may be caused by limited access of health care facilities, lack of TB diagnosis services near to villages (e.g. health posts), long distance to the nearest health facility, and lack of supervision of health workers [14]. Rural populations are also more likely to have less knowledge of TB’s symptoms and few people were aware of its severity, which contribute to the delays in diagnosis [35]. Thus, increased efforts tailored to the country’s specific circumstances are required to develop a public health system that is more responsive to the needs of patients with TB.

Concerning the appearance time of TB-related symptoms, our study showed that students who presented with PTB symptoms in the first quarter of the year were more likely to experience a diagnostic delay, which is consistent with patterns observed elsewhere in the world [36, 37]. Given the long time period between the exposure and the onset of TB, one possible explanation for the excessive number of reported cases in spring is increased transmission during winter, because both low temperature and high PM2.5 level may increase the time spent indoors with poor ventilation [38, 39]. Another possible explanation is the weakened host immunity during winter, which is associated with the high prevalence of other respiratory infectious diseases, similar symptoms and transmission routes, vitamin D deficiency due to insufficient ultraviolet radiation exposure, and high PM2.5 level, which can impair respiratory system immune response [38, 40, 41]. Therefore, they are easily confused, and this could result in health system delays. Moreover, a grand festival called the Spring Festival occurred in January or February. Patients more likely to seek medical care after this festival. In addition, the cold weather during this period may reduce patients' willingness to go to medical institutions.

Students with not available sputum-smear were more likely to have a diagnostic delay than those with negative test results. This suggests that primary health centers rely more on radiography than microbiological confirmation for diagnosis. On the other hand, it also related to the poor skill of healthcare providers in detecting the disease or poor sensitivity of diagnostic methods to detect cases at early stage. In order to obtain microbiological confirmation, patients need to go to designated hospitals, and the process of referral and tracing can cause a diagnostic delay [42]. Therefore, the laboratory testing level should be strengthened, and conventional smear culture should be combined with genetic testing to improve the detection rate of pathogens in Quzhou City in the future. In particular, medical staffs should improve the vigilance of smear-negative patients with suspicious symptoms to avoid the diagnosis delay.

The principal sources of PTB detection among students during the study period were passive case-finding, referrals, and tracing, whereas only a small proportion of PTB cases were detected through health examinations and active screening. However, patients diagnosed through health examination were less likely to experience a diagnostic delay. This is because health examinations are required before entry into higher education; thus, students and parents pay more attention to such examinations. This suggests that increased regular screening and health surveillance among students, particularly those with symptoms of coughing for more than two weeks, should be considered to avoid school PTB outbreaks [43, 44].

The current study has some strengths. First, few studies collect students’ data on the health of TB in such an area of a high prevalence of TB. we have carried out a detailed descriptive epidemiological study to provide data references for the TB control. Second, the duration of delay was based on the TBIMS, not on patient self-reporting, which greatly reduced recall bias. However, our study has limitations. First, the students’ medical information on TBIMS was limited. Therefore, the delayed decision, as the important things in delayed diagnostic, which be affected by the socioeconomic status of the family (income, education level of the parents), coverage of health care by insurance, the quality and accessibility of health service(standardized modalities, ratio of health workers, presence of health volunteer)in students were not analyzed. Second, this study was conducted in only one city in Zhejiang; hence, the generalizability of our findings is limited. In the future the combined questionnaire with routine surveillance could be useful for providing more evidence in the quality of the TB control in Quzhou.

## Conclusion

In this study, the incidence of PTB and the average annual incidence of diagnostic delay among students in Quzhou were relatively moderate. Students living in rural areas who developed TB in the first quarter of the year and diagnosed as not available sputum-smear had a greater risk of diagnostic delay, while patients among students diagnosed through health examination were at a lower risk of diagnostic delay. Therefore, schools in rural areas should pay special attention to monitoring and analyzing the incidence of TB among students in the first quarter of the year. They should also establish a system for TB prevention and control, implement morning checkups, monitor and register the causes of absenteeism due to illness, provide health education on TB prevention and control to students and staff, improve campus environmental sanitation, and strengthen ventilation in gathering places.

## Data Availability

All data generated or analysed during this study are included in this published article.

## Abbreviations

PTB: Pulmonary tuberculosis
TBIMS: Tuberculosis Information Management System
AORs: Adjusted odds ratios
COVID-19: Corona Virus Disease 2019
AFB: Acid fast bacilli

## References

[1] Bagcchi S (2023). WHO's global tuberculosis report 2022. Lancet Microbe.

[2] Jassal MS, Bishai WR (2010). Epidemiology and challenges to the elimination of global tuberculosis. Clin Infect Dis.

[3] Madebo T, Lindtjorn B. Delay in treatment of pulmonary tuberculosis: an analysis of symptom duration among Ethiopian patients. MedGenMed. 1999:E6. PMID: 11104408. 11104408

[4] SegagniLusignani L, Quaglio G, Atzori A (2013). Factors associated with patient and health care system delay in diagnosis for tuberculosis in the province of Luanda. Angola Bmc Infect Dis.

[5] Tupasi TE, Gupta R, Quelapio MID, et al. Feasibility and cost-effectiveness of treating multidrug-resistant tuberculosis: a cohort study in the Philippines. PLoS Med. 2006;3(9):e352. 10.1371/journal.pmed.0030352. 10.1371/journal.pmed.0030352 PMC1564168 16968123

[6] Rajbhandary SS, Marks SM, Bock NN. Costs of patients hospitalized for multidrug-resistant tuberculosis. Int J Tuberc Lung Dis. 2004;8(8):1012–6. PMID: 15305486. PMC5451103 15305486

[7] Chen Hui, Zhang Canyou, Zhang Hui, Cheng Jun, Li Tao (2022). Analysis of the epidemic situation of pulmonary tuberculosis in schools in China from 2004 to 2021. Chin J Antitubrec.

[8] Chen Jun, Zhao Yanlin (2021). Problem and solution for tuberculosis prevention and control in schools. Chin J Sch Health..

[9] Chen Hui, Zhang Hui, Cheng Jun (2021). guidelines for the prevention and control of tuberculosis in schools in China. Chin J Antitubrec..

[10] Wei W, Hong L, Xiaogang H. Epidemiological characteristics of tuberculosis among students in Quzhou during 2005–2012: Prevention and control. Chin J Antitubrec. 2014;35(06):866–7.

[11] Li T, Cheng Q, Li C (2019). Evidence for heterogeneity in China's progress against pulmonary tuberculosis: uneven reductions in a major center of ongoing transmission, 2005–2017. Bmc Infect Dis.

[12] National Health and Family Planning Commission of the People's Republic of China. Classification of tuberculosis WS 196-2017. Chinese Journal of Infection Control. 2018;17(04):2.

[13] Ministry of Health P. R. China: Diagnostic Criteria for Pulmonary Tuberculosis. In.: People’s Medical Publishing House; 2008.

[14] Belay M, Bjune G, Ameni G, Abebe F (2012). Diagnostic and treatment delay among tuberculosis patients in Afar Region, Ethiopia: a cross-sectional study. BMC Public Health.

[15] Li Y, Ehiri J, Tang S (2013). Factors associated with patient, and diagnostic delays in Chinese TB patients: a systematic review and meta-analysis. Bmc Med.

[16] Wang Q, Ma A, Han X (2017). Hyperglycemia is associated with increased risk of patient delay in pulmonary tuberculosis in rural areas. J Diabetes.

[17] Jia L, Jingjing Y, Ling H. Epidemiological characterization of tuberculosis patients in mobile population in Fuzhou City, 2015–2020. Applied Preventive Medicine. 2023;29(02):105–7.

[18] Jianjun L, Yanlin Z, Mingting C, Caihong X, Hui Z. Technical Guidelines for Tuberculosis Control in China. Beijing: People’s Health Publishing House; 2021.

[19] Zerbini E, Chirico MC, Salvadores B, et al. Delay in tuberculosis diagnosis and treatment in four provinces of Argentina. Int J Tuberc Lung Dis. 2008;12(1):63–8. PMID: 18173879. 18173879

[20] Sherman LF, Fujiwara PI, Cook SV, Bazerman LB, Frieden TR. Patient and health care system delays in the diagnosis and treatment of tuberculosis. Int J Tuberc Lung Dis. 1999;3(12):1088–95. PMID: 10599012. 10599012

[21] Tattevin P, Che D, Fraisse P (2012). Factors associated with patient and health care system delay in the diagnosis of tuberculosis in France. Int J Tuberc Lung Dis: Offic J Int Union Against Tuberc Lung Dis.

[22] Farah MG, Rygh JH, Steen TW, et al. Patient and health care system delays in the start of tuberculosis treatment in Norway. Bmc Infect Dis. 2006;6:33. 10.1186/1471-2334-6-33. 10.1186/1471-2334-6-33 PMC1435913 16504113

[23] Okur E, Yilmaz A, Saygi A, et al. Patterns of delays in diagnosis amongst patients with smear-positive pulmonary tuberculosis at a teaching hospital in Turkey. Clinic microbiol infect. 2006;12(1):90–2. 10.1111/j.1469-0691.2005.01302.x. 10.1111/j.1469-0691.2005.01302.x 16460554

[24] Lulu B, Hong C, Yan H, Binbing Z, Yongqin T (2021). Determinants of the delay in case-finding, treatment, and diagnosis among students tuberculosis patients in Guiyang from 2014 to 2020. Chin J Sch Health.

[25] Ying F, Jun J, Xiaolong Z, Yun L, Feixian W (2021). Delay in student pulmonary tuberculosis case-finding and associated factors in Suzhou during 2011 to 2020. Chin J Sch Health.

[26] Jie H, Weiling G, Yuanhang W (2021). Influencing factors for delay of pulmonary tuberculosis case finding in students in Jiaxing, Zhejiang, 2010–2019. Dis Surveill..

[27] Chee Cheong K, Mohd Ghazali S, Md Zamri ASS, et al. Gender differences in factors associated with the total delay in treatment of pulmonary tuberculosis patients: a cross-sectional study in Selangor, Malaysia. Int J Env Res Pub He. 2022;19(10). 10.3390/ijerph19106258 10.3390/ijerph19106258 PMC9140698 35627796

[28] Xi L, Jie L (2020). Screening and follow-up of tuberculosis among undergraduate freshmen in Haidian District, Beijing, 2011–2015. J Tuberc Lung Dis.

[29] Liu JJ, Yao HY, Liu EY. Analysis of factors affecting the epidemiology of tuberculosis in China. Int J Tuberc Lung Dis. 2005;9(4):450–4. PMID: 15830752. 15830752

[30] Yang Y, Li X, Zhou F, Jin Q, Gao L (2011). Prevalence of drug-resistant tuberculosis in mainland China: systematic review and meta-analysis. Plos One.

[31] Bogale S, Diro E, Shiferaw AM, Yenit MK (2017). Factors associated with the length of delay with tuberculosis diagnosis and treatment among adult tuberculosis patients attending at public health facilities in Gondar town, Northwest, Ethiopia. Bmc Infect Dis.

[32] Saqib SE, Ahmad MM, Amezcua-Prieto C, Virginia M (2018). Treatment delay among pulmonary tuberculosis patients within the pakistan national tuberculosis control program. Am J Trop Med Hyg.

[33] Lin Y, Enarson DA, Chiang C (2015). Patient delay in the diagnosis and treatment of tuberculosis in China: findings of case detection projects. Public health action.

[34] Gebeyehu E, Azage M, Abeje G. Factors associated with patient's delay in tuberculosis treatment in Bahir Dar City administration, Northwest Ethiopia. Biomed Res Int. 2014:701429. 10.1155/2014/701429 10.1155/2014/701429 PMC4055020 24982901

[35] Aljassim N, Ostini R (2020). Health literacy in rural and urban populations: a systematic review. Patient Educ Couns.

[36] Naranbat N, Nymadawa P, Schopfer K, Rieder HL (2009). Seasonality of tuberculosis in an Eastern-Asian country with an extreme continental climate. Eur Respir J.

[37] Willis MD, Winston CA, Heilig CM, et al. Seasonality of tuberculosis in the United States, 1993–2008. Clinic Infect Dis. 2012;54(11):1553–60. 10.1093/cid/cis235. 10.1093/cid/cis235 PMC4867465 22474225

[38] You S, Tong YW, Neoh KG, Dai Y, Wang C (2016). On the association between outdoor PM (2 5) concentration and the seasonality of tuberculosis for Beijing and Hong Kong. Environmental pollution (Barking, Essex : 1987).

[39] Tedijanto C, Hermans S, Cobelens F, Wood R, Andrews JR (2018). Drivers of seasonal variation in tuberculosis incidence: insights from a systematic review and mathematical model. Epidemiology..

[40] Yueying C, Lingwen Y, Chao L, Xi W, Jian L. Challenges and opportunities for tuberculosis prevention and control during emerging respiratory infectious disease epidemic and post era. Public Health and Preventive Medicine. 2022;33(06):6–10.

[41] Guo C, Du Y, Shen SQ (2017). Spatiotemporal analysis of tuberculosis incidence and its associated factors in mainland China. Epidemiol Infect.

[42] Yang Ni, Rao Zhengyuan, Xia Lan, Song Yang (2019). Influencing factors for delay in case-finding of active pulmonary tuberculosis in students in Sichuan, 2016–2018. Dis Surveill.

[43] Xu J, Wang G, Zhang Y (2019). An outbreak of tuberculosis in a middle school in Henan, China: Epidemiology and risk factors. Plos One..

[44] Hou J, Pang Y, Yang X (2020). Outbreak of mycobacterium tuberculosis Beijing Strain in a High School in Yunnan, China. Am J Trop Med Hyg.

